# Do drug package inserts meet the rules and regulations of Iran’s Food and Drug Administration in terms of informing patients?

**DOI:** 10.15171/hpp.2019.30

**Published:** 2019-08-06

**Authors:** Tahereh Eteraf-Oskouei, Saeid Abdollahpour, Moslem Najafi, Vahideh Zarea Gavgani

**Affiliations:** ^1^Department of Pharmacology & Toxicology, Faculty of Pharmacy, Tabriz University of Medical Sciences, Tabriz, Iran; ^2^Department of Medical Library and information Science, School of Management and Medical Informatics, Tabriz University of Medical Sciences, Tabriz, Iran; ^3^Tabriz Health Services Management Research Center, Tabriz University of Medical Sciences, Tabriz, Iran

**Keywords:** Health Communication, Package inserts, Drug product, Drug labeling, Neurologic, Psychiatric, Medication

## Abstract

**Background:** Drug package inserts (PIs) are the most accessible source of information for users and are designed to aid the safe use of medicines and avert adverse events. This study measured the conformity of PIs with the health communications standards of Iran’s Food and drug Administration (FDA).

**Methods: ** This descriptive cross-sectional study evaluated 92 PIs related to 22 best-selling neurological and psychiatric drugs in Iran based on criteria approved by Iran’s FDA. Six categories of criteria were considered in evaluating the extent of conformity: I) writing and formatting, II) references, III) drug description, IV) warnings and precautions, V) interactions, and VI) side effects. Each PI was scored based on observation of standards; data was analyzed using Microsoft Excel pivot tables.

**Results: ** In total, 2929 items from 92 PIs were evaluated, of which 37 (40.2%) were related to antidepressants, 31 (33.7%) to sedatives and hypnotics, and 24 (26%) to anticonvulsant drugs. The PI content was insufficient in various aspects of conformity with standards in each category. Among the six categories, the best match was found in warnings and precautions with 667items (72.5%), followed by writing and formatting with 663 (69.1%). The lowest conformity was found in the reference category with 194 (26.4%) items.

**Conclusion: ** The PIs of Iranian neurological drugs do not fully meet Iran’s FDA standards. It is strongly recommended that smart PIs be developed using mobile apps to overcome this problem.

## Introduction

The main purpose of drug package inserts (PIs) is to disseminate essential information on medication among patients and consumers to aid the safe use of drugs and enhance health choices in line with health communication. Health communication is defined as the “use of methods to inform and influence individual and community decisions that enhance health”.^1^ “Health communication provides a research-based foundation for developing strategies to inform and influence individual and community health decisions”.^2^ The intention of health communication is to enhance personal health choices by improving health literacy. The rational use of drugs is one of the eight principles of primary health care, and one of its requirements is public education regarding the correct use of drugs.^3^ The promotion of patients’ knowledge about drugs is an important issue due to the rise in self-medication, medication errors, and drug abuse. Therefore, patients need to learn about the effects, interactions, use, precautions, and side effects of prescribed medicines in order to avoid medicating errors and to control the promotion of health.

Errors commonly occur in hospitals, homes, and outpatient settings throughout all steps of the medication process, i.e. prescribing, dispensing, administering, and monitoring the patient’s response. Research has shown that drug packaging and labeling confusions are involved in more than 33% of all medication errors.^4^ In hospitals, “medication errors occur most frequently at the prescribing and administration stages”.^5^ The most common errors that occur in the dispensing, labeling, and administration of medication use may be easily avoided by health communication through single PIs.

The liability for patient safety and health communication requires medication manufacturers to provide informative and evidence-based content on PIs in packaging medicinal productions. The FDA monitors the rules and regulations of PIs to ensure that the essential information is communicated to consumers. If these brochures are designed on sound principles and made understandable for patients of any social class and educational level, the likely result will be more rational use of drugs and reduced self-medication, one of the most important causes of drug side effects.^6^

The PI is the first consult with patients and medicine consumers, and in some countries, PIs are known as the main source of drug information.^7^ Evidence shows, however, that consumers and health professionals do not always find the PIs to be appropriate, informative, or responsive,^8^ and they have a variety of problems, such as reading ease, responsiveness, and not being evidence-based. If pharmaceutical manufacturers do not commit to meeting the criteria of food and drug regulations and providing appropriate content, patient health and safety may be compromised by PIs. Few studies have been done about the characteristics of PIs of Iranian pharmaceutical manufacturers.^9^ The only related study found was Zarghami et al’s study that examined 34 psychiatric drugs made by 29 Iranian pharmaceutical companies based on a set of self-identified criteria collected from the literature.^6^ Consequently, the current literature review shows that there is a research gap in the potential evaluation of Iranian PIs for health communication. Thus, this study was conducted to evaluate the compliance of PIs for best-selling neurological and psychiatric medicines with the guidelines and regulations of Iran’s FDA regarding patient informing.

## Materials and Methods

This descriptive and cross-sectional study was conducted on 92 PIs of 22 best-selling neurological and psychiatric medicines manufactured by 31 pharmaceutical companies in Iran. The best-selling medicines were identified through the website of the Iran FDA, affiliated with the Ministry of Health and Medical Education, and through consultation with the Deputy of Drug and Food of the province of East Azerbaijan in Tabriz city, Iran. The inclusion criteria for the PIs were: (1) best-selling medicines, (2) oral medication, (3) produced in Iran, and (4), listed in the subgroup of neurology and psychiatry (anticonvulsants, antidepressants, sedatives, and hypnotics).

### Data collection

Data was collected from the PIs of the identified medicines, as there is no official information source for Iranian FDA-approved PIs similar to Daily Med,^10^ a databank of FDA-approved drug PIs provided by the US government. The names of drug manufacturing companies and the brand names of drugs were determined through a search of “Darooyab”,^11^ and the pharmacies carrying each medicine were identified in order to access the related PIs. Some PIs were available through the manufacturer’s website, but the PIs on the website differed from those in the drug packages. Therefore, based on the list obtained from Darooyab, PIs were collected from pharmacies throughout Tabriz, Ardebil, and Meshginshahr, the largest cities in East Azerbaijan province. The drugs for which PIs were collected are listed in Table 1. In total, 92 PIs were collected, 31, 37, and 24 of which were related to best-selling hypnotic and sedative, antidepressant, and anticonvulsant medicines, respectively. All PIs under study were divided into three categories: (1) sedatives and hypnotics; (2) antidepressants; and (3) anticonvulsants.

The standards for the development of PIs were obtained through the website of the Ministry of Health and Medical Education,^12^ and all PIs were evaluated based on that criteria. Among the standards, 52 items were included in this evaluation on the basis of relevancy to patient (drug utilizer) information, with the approval of the maximum votes of a panel of five experts who had work experience in the FDA, three of whom were faculty members of the pharmacy department and health information specialists. In Iran, a medication guide is intended to inform all consumers, including physicians, pharmacists, and patients. For the purposes of this study, those items more related to patients were selected. The selected criteria were classified into six categories (writing and formatting, drug descriptions, warnings and precautions, interactions, side effects, and references) consisting of 13, 8, 10, 4, 7, and 10 items available in the FDA standards and guidelines, respectively (52 items in total).

Furthermore, the standards of the medication guide consist of six general statements that pharmaceutical companies are required to mention in the PIs. These general statements are:

General statement 1: The doctor will determine the dose of each medicine; however, the usual dose of medicine is as follows;

General statement 2: This medicine is prescribed for your current condition, so avoid using it in similar cases, and never offer this medicine to others;

General statement 3: If you take other medicines, be sure to inform your doctor;

General statement 4: Any medication may cause some unwanted adverse effects along with therapeutic effects, though not all of these adverse effects are seen in an individual. Consult your doctor if any adverse effects occur;

General statement 5: If you accidentally use more than the recommended dose, call your doctor or a treatment center immediately; and General statement 6: If side effects other than those in the medication guide occur, you can contact your doctor, pharmacist, or treatment center.

These general statements were categorized and examined based on subject congruity among the components of the six categories. For example, general statement (1) was examined considering the description of the medicine; general statement (2) for warnings and precautions; general statement (3) for drug interactions; and general statements (4), (5), and (6) were examined considering adverse effects of the drug.

PIs were rated to obtain quantitative information for comparison. Scores of 0 to100 were used for each criterion with a score of one hundred being considered for each criterion observed in a PI, and zero for each criterion not observed in a PI. Finally, the mean percentage was obtained for each PI as well as each criterion. To calculate the compliance of the information provided in the PIs with the FDA standards in terms of the drug and drug groups, the number of PIs examined for each drug was multiplied by 52 and placed in the denominator of a fraction. The number of compliances was then placed in the numerator of the fraction. To calculate the percentage of total compliance, the sum of the compliances of the same group was placed in the numerator, and the sum of PIs studied which belonged to the same group was multiplied by 52 and placed in the denominator of the fraction; the number obtained was then multiplied by 100. The evaluation was repeated twice by two evaluators on the research team. Microsoft Excel software 16 was used for statistical analysis.

## Results

In total, 92 PIs from 31 pharmaceutical companies were evaluated in this study. The PIs were related to 22 neurological and psychiatric medicines. The PIs were evaluated on six aspects based on the protocol: writing and formatting, drug descriptions, warnings and precautions, interactions, side effects, and references. The results of this study showed that more than 50% of the standards under study were observed in the six categories of all PIs related to medicines for neurology and psychiatry. Approximately 62.03% of the PIs for the medicines in the sedatives and hypnotics drug group complied with the standards. In contrast, anticonvulsants had the lowest rate of conformity (59.29%) with the standards. Among the medicines in the sedatives and hypnotics group, chlordiazepoxide had the highest rate of compliance with the standards (approximately 67%), and phenobarbital had the lowest rate of compliance (56%). In the anticonvulsants group, phenytoin had the lowest rate of compliance (approximately 52%), and gabapentin had the highest compliance rate (61.5%). Among antidepressants, fluoxetine had the highest rate of compliance with the standards (69%), and the PI of clomipramine had the lowest compliance rate (53.8%) (Table 1).

**Table 1:** Compliance of PIs with FDA standards by medicinal group

**Medicinal group**	**Generic name**	**Number of PIs**	**Number of conformities**	** % f conformity**	**Total number of compliances in category**	**% Of conformity in category**
Sedatives and Hypnotics	Diazepam	2	64	61.5	1000	62.03
	Alprazolam	6	197	63.1		
	Zolpidem	9	295	63		
	Phenobarbital	3	88	56.4		
	Clonazepam	4	124	59.6		
	Lorazepam	4	127	61.1		
	Chlordiazepoxide	3	105	67.3		
Antidepressants	Amitriptyline	2	64	61.5	1189	61.79
	Imipramine	3	91	58.3		
	Desipramine	2	61	58.7		
	Doxepin	2	64	61.5		
	Sertraline	7	228	62.6		
	Citalopram	8	244	58.7		
	Fluoxetine	4	144	69.2		
	Fluvoxamine	4	137	65.9		
	Clomipramine	2	56	53.8		
	Nortriptyline	3	100	64.1		
Anticonvulsants	Phenytoin	2	54	51.9	740	59.29
	Lamotrigine	3	89	57.1		
	Valproate	5	154	59.2		
	Carbamazepine	4	123	59.1		
	Gabapentin	10	320	61.5		
**Total**		**92**	**2929**	**61.22**		

Details of the findings are shown in Tables 2 and 3 based on each of the six categories, i.e. writing and formatting (10 items), drug descriptions (13 items), warnings and precautions (10 items), interactions (4 items), side effects (7 Items), and references (8 items). Table 2 shows a complete schema for the criteria investigated in each of the six categories. Table 3 shows the state of compliance according to the drug group (sedatives and hypnotics, antidepressants, and anticonvulsants) in each of the six categories.

**Table 2:** Conformity of PIs with FDA standards according to the defined criteria

**Category**	**Total**	**Criterion**	**No.**	**Percent**
Writing and formatting	636 (69.1%)	Persian language	92	100
		English language	30	32.6
		Scientific terms without explanation	51	55.4
		Abbreviations without explanation	80	86.9
		Punctuation	92	100
		Braille alphabet	0	0
		Foldable paper	92	100
		Contrast	92	100
		Titles being highlighted by bold font	92	100
		No. of titles	15	16.3
Drug description	824 (68.9%)	Iran drug list (IDL)	92	100
		Dose and administration	89	96.7
		Specific age groups	66	71.7
		General statement 1	67	72.8
		Dosage forms	83	90.2
		Brand name	25	27.2
		Generic name	92	100
		Amount of effective ingredient	81	88.0
		How supplied	39	42.4
		Indications	47	51.1
		Drug class	14	15.2
		Non-exaggeration	92	100
		Contraindications	37	40.2
Interactions	245 (66.6%)	Drug interactions	32	34.8
		General statement 3	78	84.8
		Taking medicine with food	54	58.7
		Impact on behavior	81	88.0
Reference	194 (26.4%)	Including references list	24	26.1
		Revision date	11	11.9
		Address	67	72.8
		Telephone	34	36.9
		Email	24	26.1
		Website/URL	29	31.5
		The priority of reference appearance	0	0
		Reference of each paragraph	5	5.4
Warning and precautions	667 (72.5%)	General statement 2	84	91.3
		Points related to special groups	88	95.7
		Pregnancy and breastfeeding	92	100
		Different color and font in contraindications in pregnancy	56	60.9
		Warnings	58	63.0
		Different color and font of warning	1	1.1
		Imperative statements in warning	52	56.5
		Not using the medicine with expired date	61	66.3
		Keeping out of reach of children	84	91.3
		Keeping conditions	91	98.9
Side effects	363 (56.4 %)	Mentioning side effects	92	100
		General statement 4	76	82.6
		Common and rare adverse effects	45	48.9
		General statement 5	41	44.6
		Adverse effects that require consulting the doctor	65	70.7
		General statement 6	7	7.6
		Signs of poisoning	37	40.2

**Table 3:** Conformity of criteria presented in PIs with FDA standards by medication groups, separately

**Criteria**	**Drug Group**		
	**Anticonvulsant**	**Antidepressant**	**Sedative & Hypnotic**
	**No. (%)**	**No. (%)**	**No. (%)**
Side effects	88 (52.4)	148 (57.1)	127 (58.5)
Interactions	60 (62.5)	109 (73.6)	76 (61.3)
Warnings & precautions	170 (70.8)	276 (74.6)	221 (71.3)
Drug description	209 (66.9)	326 (67.8)	289 (71.7)
References	44 (22.9)	80 (27.0)	70 (28.2)
Writing & formatting	169 (70.4)	250 (67.6)	217 (70.0)

### Writing and formatting

This study indicated that all PIs were in the local language (Persian); the content information of almost one-third of PIs was also given in English. The appearance features of PIs, such as paper, contrast between text and background, font, and print, were very good, but not suitable in terms of clarity or simplicity. About 87% of the PIs had specialized abbreviations with no explanations, and more than half of them had 55.4% new scientific terminology with no explanation. None of the PIs had information regarding warnings, interactions, side effects, references, or drug descriptions written in Braille for the blind (Table 2). Regarding the principles of writing, the antidepressants drug group complied on 250 items (67.6%), giving it a more satisfactory position than the sedatives and anticonvulsants group concerning compliance with FDA standards (Table 3).

### Drug description 

The FDA criteria for describing a drug that were observed in all 92 studied PIs (100%) were the official list of drugs, the generic name of the drug, and the principle of non-exaggeration. In contrast, the drug group and brand name were mentioned in very few cases. Indications and contraindications were mentioned in 40-50% of cases. The dose and administration were mentioned in more than 80% of cases (Table 2). On average, 64 cases (68.8%) of drug description complied with the standards (Table 1). Furthermore, the antidepressant drug group was in better compliance with the standards regarding drug description than the sedatives and anticonvulsants groups (67.8%; 326 cases) (Table 3).

### Warnings and precautions

Warnings concerning contraindications during pregnancy and breastfeeding were clearly stated in all PIs. Storage, keeping out of reach of children, and the warning regarding use in specific populations (General statement 2: “This medicine has been prescribed for your current condition, so avoid using it in similar cases or offering this medicine to others.”) were observed in more than 90% of cases; however, only a minority of PIs had highlighted the warnings in different colors, and only about half of them had provided the warnings in imperative sentences. The status of compliance of PIs with the standards regarding warnings and precautions was better in the antidepressant drug group than in the other two groups of anticonvulsants and sedatives (74.6%; 276 cases) (Table 3).

### Interactions

Overall, the criteria regarding interactions were observed in 66.6% of cases. Food interactions were included in almost half of the PIs; however, the probable impact on an individual’s behavior, like dizziness and drowsiness, was stated more than others. A general warning about the need to provide feedback to your doctor about using other medications (general statement 3: “If you take other medicines, make sure to inform your doctor.”) was stated in almost 85% of cases (Table 2). The status of compliance of the PIs with the criteria concerning interactions was better in the antidepressant drug group than in the anticonvulsants and sedatives groups (109 cases; 73.6%). The anticonvulsants group had the weakest status of all (60 cases; 62.5%) (Table 3).

### Side effects of the medicine

The term “side effects” was included in all PIs, yet the criteria defining this indicator was included in different proportions. Likewise, general warnings (general statement 4: “Besides the therapeutic effect, each medicine may cause some unwanted adverse effects, although not all of these side effects are seen in an individual. Consult your doctor if you have any side effects.”) was included in most PIs. In contrast, general warnings (general statement 6: “In case you experience side effects other than what is stated in the medication guide, please contact your doctor, pharmacist, or treatment center.”) were mentioned in only about 7% of PIs (Table 2). A phone number was not provided for users to call for extra information, if needed. Even the standards regarding the signs of drug poisoning, common and rare side effects, drug overdose, and the related general statement (general statement 5: “If you accidentally use more than the recommended dose, refer to a doctor or a health center immediately.”) were observed in few cases. The adverse effects of medicines were written in various proportions in nearly half of the PIs (Table 2). The PIs of the antidepressant drug group had a higher status of compliance than the anticonvulsants and sedatives groups (148 cases; 57.1%), but the anticonvulsants group met fewer standards than the others (88 cases; 52.4%) (Table 3).

### 
References


In the category of references, the following evaluation indicators were considered: mentioning the reference for the contents of the PIs, the latest revision date, links and URL to the webpage containing information, and the address of the pharmaceutical manufacturing company. The findings showed that less than one-third of the PIs had mentioned the references; the list of references was provided only at the end of PIs without prioritizing and specifying which part of the information relates to which of the references. URL for website and e-mail addresses were included in about one-third of the PIs. In the PIs under study, the address of the pharmaceutical company was most often mentioned, and this feature was the only criterion with the highest score of compliance regarding references. Updating the date of the contents of the drug information of PIs was less considered, as only 11% of PIs had an updating date (Table 2). In the references category, the status of PI compliance with the criteria was higher in the antidepressant drug group (80 cases; 27.0%) than in the groups of anticonvulsants and sedatives (44 cases; 22.9% and 70 cases; 28.2%, respectively) (Table 3).


Based on the current findings, it can be concluded that among all PIs and all six categories, the criteria of warnings and precautions and references had the highest and the lowest rates of compliance with Iran FDA standards, respectively (72.5% and 26%, respectively) (Table 2). Moreover, the anticonvulsants and antidepressants drug groups had the lowest and highest rates of compliance with the standards, respectively. The PIs of all drug groups observed the drug description category more than the others and the references category less than the others (Table 3).

## Discussion

This study investigated the contribution of PIs in health communication for best-selling neurological and psychiatric medicines through their compliance with the standards of Iran’s FDA. The 52 criteria were classified into the six categories of writing and formatting, drug description, warnings and precautions, interactions, side effects, and references.

### Writing and formatting

Each of the categories in the PIs was provided in Persian/Farsi, the official language of Iran, and a small number of medicines had a bilingual PI (Persian and English). While strategic planning for effective health communication includes taking appropriate steps to analyze and segment target audiences,^13^ it is important to consider who the desired audience is, which languages the population uses, and what level of literacy the people have.

In addition, drug administration systems in multilingual countries oblige pharmaceutical companies to provide multilingual guidelines. For example, the European Union has a multilingual system which implements a project called PILLS (Patient Information Language Localization System), under which pharmaceutical companies are required to print PIs in several languages.^14^ Based on the regulations and protocol of Iran’s FDA, the Persian language is required in all PIs, and other languages are permitted as well. This means that there is no obligation for drug manufacturing factories to provide the content of PIs in multiple languages.

The findings of the current study showed that PIs are difficult to understand for general public/non-specialists, because they use technical terminology and abbreviations without referring to the full term. This finding was consistent with previous studies that evaluated the readability of PIs using the Flesch-Dayani Reading Ease scale. The study found that the readability of PIs in Iran was low; 70.9% of the PIs had a readability of 10 to 11, i.e., fairly difficult to very difficult.^15^ A field study in Germany investigated patients’ views about PIs. More than 50% of the participants stated that PIs were very difficult and should be written in such a way that it would be easy to find the required information and understand it easily.^16^ In a study that reviewed patients’ information leaflets at a district general hospital of England, the medical data provided in leaflets was higher than grade 8 (the standard reading level).^17^ In the United States, despite the existence of FDA standards and various revisions published to update them, and despite the learned intermediary doctrine in the American pharmacy system, studies have shown that the level of readability is above number 10, i.e., hard to understand.^18^

In the PIs under study, there was no support for people with visual disabilities. None of the PIs had any information in Braille. According to the pharmaceutical commission for the EU member states, providing the name of and information on the medicine in Braille is necessary for all medicine packages.^19^ However, in the United States, although factories are encouraged to provide information in Braille, the FDA has not made it obligatory, and only a few OTCs in some states provide the name of the medicine in Braille.^20^ It seems that similarly, the Iranian FDA’s policy regarding including information in Braille is encouraging, but not mandatory.

Accessibility to patient-centered PIs offered in understandable, simple, and multiple languages for the benefit of patients with different languages and levels of education is an effective and ethical provision of a medicinal guide.^14^ Iran is a country with dialects and languages other than Persian, and it seems necessary to provide PIs in languages other than Persian (the official language of Iran). According to recent studies, the highest number of medical tourists in Iran comes from Azerbaijan^21^ the speaking medium of which is Turkish, which is the second most widely spoken language in Iran as well. Therefore, this study shows a gap in Iran’s FDA regulations regarding appropriate health communication in terms of analyzing and segmenting the language characteristics and physical disabilities of target audiences as well as in the PIs. Drug manufacturing companies in Iran would do better to communicate the PIs’ information in Turkish and English in addition to Persian.

### Drug description

The current study showed that compliance of PIs with Iranian FDA standards is best in the category of drug description. The criteria of drug dose and administration, how to store, how to use, and how to supply were among the informative and fully-explained items. However, the brand name of the drug was not given the same attention by the packagers. Based on these findings, it can be stated that the information regarding rational use of medicine meets the strategies of health communication in the category of drug description.

### 
References


According to the FDA regulations, a list of references should be included in all PIs. In addition, links to more information through the URL to the website of the pharmaceutical company as well as the manufacturer’s telephone number, email address, and mailing/street address should be included in the PIs for urgent contact and referral, if necessary. Moreover, because information is updated and scientific changes in medications occur regularly, the revision date of the information should necessarily be included in the PIs. If the user wants to track a particular paragraph or specific section of the information present on the medication guide by referring to the original reference, the PIs will not meet the patient’s or caregiver’s information needs. Therefore, Iran’s FDA and other relevant organizations seem to need more monitoring of the medication guides that packagers place inside the boxes.

### Drug interaction

Presenting information regarding drug interactions is one of the most important steps in health communication through PIs. The current study indicated that interactions were mentioned in less than 35% of PIs. Nevertheless, generally speaking, of the four criteria related to interactions (direct mention of phrases related to interactions in the PIs, general statement 3, food and drug interactions, and the effect of drug interactions on behavior), the PI compliance rate with the standards and regulations of Iran’s FDA was only 66%. Previous relevant studies in other countries have shown a similar situation.

The results of a study by Koizumi et al demonstrated that information on drug interactions is not always fully presented in the medication guidelines of Japan, the United States, or the United Kingdom.^22^ Studies show that all PIs in Germany contained information on the structure and dose of the medicine; however, some of them provided instructions only on a milligram of active ingredient, rather than a single dose, such as two pills or a capsule. Furthermore, some PIs had unusual statements as instructions for use, for example, two to four pills once or three times a day.^23^

### Warnings and precautions 

Another important step in an effective health communication strategy is to review background information to define the problem (What’s out there?).^5^ The six general statements about warning and precautions are background information that all drug manufacturing companies must mention in PIs. The current study showed that all six general statements under study (see methodology) had not been considered equally in PIs. The warning to avoid medicine use in similar cases or offering this medicine to others was the most frequent warning included in the medication guide. Warnings referring to a doctor, pharmacist, or references were taken into account. For example, the statement, “If side effects other than those in the medication guide occur, you can contact your doctor, pharmacist, or treatment center,” was seen in few PIs and less frequently than other warnings. Similarly, the warning about overuse (“If you accidentally use more than the recommended dose, refer to a doctor or a health center immediately.”) was included less frequently in PIs. Warnings concerning dose and drug interactions were included in almost all medication guides. No consistency considering warnings and general statements was found among PIs produced by different companies. A similar study was found in gray literature on Iran’s best-selling medicines. Contrary to the current results, that study found that warnings were reported in almost 50% of cases.^9^

Analysis of the findings of the current study showed that nearly 50% of cases met the standards; in previous studies, the overall compliance was less. For instance, Sukkari et al^24^ investigated Arabic PIs with keystone criteria of the United States and found that only 30% of the PIs produced complied with the standard in question. A study published in Nepal on PIs advertising medicines revealed that the pharmaceutical and advertising PIs did not comply with the standards of the WHO; in most cases, the health profile of the medicine, such as side effects or adverse effects of medicine, precautions and drug interactions, were ignored.^25^ The fact is that the status of providing PIs is unfavorable throughout the world.

The accuracy of the information provided in PIs can help minimize adverse effects caused by pharmaceutical errors such as wrong dosage or lack of warning signs. Patients’ familiarity with the medicine, its benefits, and its side effects as a prerequisite of medicinal treatment will be effective and safe.^26^

Flawed and incorrect information may lead to dangerous results, such as disability and death. A good PI needs to contain confirmed, necessary, and correct information about the medicine, and it must be written in a language that would not be false or misleading advertising. PIs are based on evidence and should be updated in line with the development of clinical and para-clinical information.^7^

## Conclusion

Based on the results of the current study, it can be concluded that the promotional health information on the safe and effective use of a particular medicine was not completely standardized nor appropriately communicated in the PIs of Iran’s domestic neurological and psychiatric medications. Considering the present and previous findings in different countries, it is strongly suggested that effective strategies be developed in the FDA rules and regulation for PIs to ensure that reliable, updated, and responsive health communication is delivered to consumers through PIs. In addition, Iran is a common medical tourism destination among its neighboring countries; therefore, PIs need to be provided in multiple languages (especially Turkish) to serve the large Azerbaijani and Turk populations who come for treatment in Iran. There is no guarantee or obligation for medication manufacturers to strictly adhere to the standards in providing PIs; thus, the best solution would be to use the advantages of mobile phone applications. It is suggested that a Smart PIs App be designed to provide useful, effective, and responsive PIs so that all information is selected, created, and pre-tested based on patients’ information needs, literacy levels, and communication channels. The Smart PIs App would be customizable with a variety of language, literacy, and audio-visual options to achieve the expected outcomes of health communication. This may ensure that the problem of inconsistency in adhering to standards and providing patients with necessary, understandable, and evidence-based information about medicines is minimized.

## Ethical approval

Ethical approval was obtained from the Ethics Committee of the Tabriz University of Medical Sciences (Ethics Code: 2821). No names concerning any pharmaceutical companies have been revealed in this study.

## Competing interests

The authors declare that they have no competing interests.

## Funding

None.

## Authors’ contributions

Conceptualization, designing, writing and revision: VZ and TE; data collection and drafting: SA and MN. Supervision: VZ, TE and MN. Statistics: SA and AJ.

## Acknowledgments

The authors would like to express their appreciation to the pharmacists, managers, and technical officers working in the pharmacies of Tabriz, Meshginshahr, and Ardebil for their assistance in accessing pharmaceutical PIs. We would also expand our appreciation to Dr. Ali Jafari for his help in statistics analysis.

## References

[1] Freimuth VS, Quinn SC (2004). The contributions of health communication to eliminating health disparities. Am J Public Health.

[2] Beato RR, Telfer J (2010). Communication as an essential component of environmental health science. J Environ Health.

[3] Keyvanara M, Safaeian L, Karimi S, Shojaiezadeh N (2016). Rational use and prescription of drugs: a review on WHO’s 12 strategies. Hakim Health System Research.

[4] Jeetu G, Girish T (2010). Prescription drug labeling medication errors: a big deal for pharmacists. J Young Pharm.

[5] Aspden P, Wolcott JA, Bootman JL, Cronenwett LR (2007). Preventing Medication Errors.

[6] Zarghami M, Azari A, Ghasemi S, Hormozpour M, Hendouei N (2016). Availability of drug key information on package inserts of psychiatric drugs manufactured in Iranian pharmaceutical companies. Iran J Psychiatry Clin Psychol.

[7] Ramdas D, Chakraborty A, Hs S, Faizan S, Kumar VP, Bn S (2013). A study of package inserts in southern India. J Clin Diagn Res.

[8] Sawalha A, Sweileh W, Zyoud S, Jabi S (2008). Comparative analysis of patient package inserts of local and imported anti-infective agents in Palestine. Libyan J Med.

[9] Mirzadeh-Qasabeh S (2018). Comparative review of package inserts with the criteria of package inserts of Iran’s Food and Drug Organization and US Food and Drug Administration Medical Library and Information Sciences.

[10] Dailymed 2019. Available from: https://dailymed.nlm.nih.gov/. Accessed July 23, 2019.

[11] Darooyab 2019. Available from: http://www.darooyab.ir/. Accessed July 23, 2019.

[12] KAUMS 2019. Available from: http://fdo.kaums.ac.ir/Default.aspx?PageID=90. Accessed July 23, 2019.

[13] Health Communication Basics: What is health communication: Centers for Disease Control and Prevention. Available from: https://www.cdc.gov/healthcommunication/healthbasics/WhatIsHC.html. Updated 4 March 2019. Accessed July 23, 2019.

[14] Bouayad-Agha N, Power R, Scott D, Belz A. PILLS: Multilingual generation of medical information documents with overlapping content. University of Brighton; 2002. Available from: http://mcs.open.ac.uk/nlg/old_projects/wysiwym/papers/ITRI-02-04.pdf. Accessed July 23, 2019.

[15] Zarea Gavgani V, Mirzadeh-Qasabeh S, Hanaee J, Hamishehkar H (2018). Calculating reading ease score of patient package inserts in Iran. Drug Healthc Patient Saf.

[16] Fuchs J, Hippius M, Schaefer M (2005). A survey of package inserts use by patients. Hospital Pharmacy Europe.

[17] Williamson JM, Martin AG (2010). Analysis of patient information leaflets provided by a district general hospital by the Flesch and Flesch-Kincaid method. Int J Clin Pract.

[18] Basara LR, Juergens JP (1994). Patient package insert readability and design. Am Pharm.

[19] European Commission. Guideline on the readability of the label and package leaflet of medicinal products for human use. Brussels, Belgium: European Commission; 1998.

[20] Botta M. Braille in Pharma: Differing Views in the U.S. and Europe. Pharmaceutical Processing; 2017. Available from: https://www.rdmag.com/article/2017/09/braille-pharma-differing-views-us-and-europe. Accessed July 23, 2019.

[21] Momeni K, Janati A, Imani A, Khodayari-Zarnaq R (2018). Barriers to the development of medical tourism in East Azerbaijan province, Iran: A qualitative study. Tour Manag.

[22] Hirata-Koizumi M, Saito M, Miyake S, Hasegawa R (2007). Adverse events caused by drug interactions involving glucuronoconjugates of zidovudine, valproic acid and lamotrigine, and analysis of how such potential events are discussed in package inserts of Japan, UK and USA. J Clin Pharm Ther.

[23] Fuchs J, Hippius M, Schaefer M (2006). Analysis of German package inserts. Int J Clin Pharmacol Ther.

[24] Sukkari SR, Al Humaidan AS, Sasich LD (2012). The usefulness and scientific accuracy of private sector Arabic language patient drug information leaflets. Saudi Pharm J.

[25] Sah P, Sah AK, Jha RK (2012). Evaluation of the rationality of psychotropic drug promotional literatures in Nepal. J Drug Deliv Ther.

[26] Raynor DK, Blenkinsopp A, Knapp P, Grime J, Nicolson DJ, Pollock K (2007). A systematic review of quantitative and qualitative research on the role and effectiveness of written information available to patients about individual medicines. Health Technol Assess.

